# Post-Transcriptional Dysregulation by miRNAs Is Implicated in the Pathogenesis of Gastrointestinal Stromal Tumor [GIST]

**DOI:** 10.1371/journal.pone.0064102

**Published:** 2013-05-24

**Authors:** Lorna Kelly, Kenneth Bryan, Su Young Kim, Katherine A. Janeway, J. Keith Killian, Hans-Ulrich Schildhaus, Markku Miettinen, Lee Helman, Paul S. Meltzer, Matt van de Rijn, Maria Debiec-Rychter, Maureen O’Sullivan, NIH Pediatric and wild-type GIST Clinic

**Affiliations:** 1 Histopathology Department, School of Medicine, Trinity College Dublin, Dublin, Ireland; 2 National Children’s Research Centre, Our Lady’s Children’s Hospital, Crumlin, Dublin, Ireland; 3 Computational Biology, Systems Biology/Immunology, Animal and Grassland Research and Innovation Centre, Teagasc, Dunsany, County Meath, Ireland; 4 Centre for Cancer Research, National Cancer Institute, Bethesda, Maryland, United States of America; 5 Department of Pediatric Hematology-Oncology, Dana Farber Cancer Institute and Children’s Hospital, Boston, Massachusetts, United States of America; 6 Institute of Pathology, University of Cologne Medical Centre, Cologne, Germany; 7 Department of Pathology, Stanford University Medical Centre, Stanford, California, United States of America; 8 Department of Human Genetics, Catholic University Leuven and University Hospitals, Leuven, Belgium; Institute of Pathology, Germany

## Abstract

In contrast to adult mutant gastrointestinal stromal tumors [GISTs], pediatric/wild-type GISTs remain poorly understood overall, given their lack of oncogenic activating tyrosine kinase mutations. These GISTs, with a predilection for gastric origin in female patients, show limited response to therapy with tyrosine kinase inhibitors and generally pursue a more indolent course, but still may prove fatal. Defective cellular respiration appears to underpin tumor development in these wild-type cases, which as a group lack expression of succinate dehydrogenase [SDH] B, a surrogate marker for respiratory chain metabolism. Yet, only a small subset of the wild-type tumors show mutations in the genes coding for the SDH subunits [SDHx]. To explore additional pathogenetic mechanisms in these wild-type GISTs, we elected to investigate post-transcriptional regulation of these tumors by conducting microRNA (miRNA) profiling of a mixed cohort of 73 cases including 18 gastric pediatric wild-type, 25 (20 gastric, 4 small bowel and 1 retroperitoneal) adult wild-type GISTs and 30 gastric adult mutant GISTs. By this approach we have identified distinct signatures for GIST subtypes which correlate tightly with clinico-pathological parameters. A cluster of miRNAs on 14q32 show strikingly different expression patterns amongst GISTs, a finding which appears to be explained at least in part by differential allelic methylation of this imprinted region. Small bowel and retroperitoneal wild-type GISTs segregate with adult mutant GISTs and express SDHB, while adult wild-type gastric GISTs are dispersed amongst adult mutant and pediatric wild-type cases, clustering in this situation on the basis of SDHB expression. Interestingly, global methylation analysis has recently similarly demonstrated that these wild-type, SDHB-immunonegative tumors show a distinct pattern compared with KIT and PDGFRA mutant tumors, which as a rule *do* express SDHB. All cases with Carney triad within our cohort cluster together tightly.

## Introduction

Gastrointestinal stromal tumor (GIST) is the commonest sarcoma of the gastrointestinal tract, typically presenting clinically in patients aged 55–65 years [1]. Classically, GISTs are characterised by activating mutations in the genes encoding the type III tyrosine kinase receptors, *KIT* [2] occurring in ∼80–85%, or *Platelet-Derived Growth Factor Receptor, alpha PDGFRA* [3], in 5–8% of GISTs [1]. These mutually exclusive mutations cause ligand-independent auto-phosphorylation of the receptor, activating crucial growth and survival signalling cascades. Rare GISTs, lacking *KIT* and *PDGFRA* mutations, have been found to contain a common *BRAF* exon 15 activating mutation resulting in a V600E substitution [4]. The 10–15% of GISTs with no detectable *KIT*, *PDGFRA* or *BRAF* mutations have been termed ‘wild-type’ (WT) GISTs. WT GISTs are generally KIT immunopositive [5] and have similar downstream signalling to mutant tumors, despite the lack of activating mutations [5]. The majority of pediatric GISTs are WT, typically presenting as slow-growing gastric tumors in prepubescent girls.

Additional key differences between adult and pediatric GIST include large-scale genomic losses of chromosomes 14q, 22q, 1p and 9p with disease progression in adult tumors [6], changes which are mostly absent in pediatric GISTs or in tumors associated with Carney Triad and Carney-Stratakis syndromes [7]. Differences in mRNA expression profiles between adult and pediatric GISTs have also been reported [8], [9]. WT GIST may be associated with a number of syndromes including Neurofibromatosis type-1 (NF-1), Carney triad and Carney-Stratakis syndrome (or Carney dyad). Carney triad describes the non-heritable association of GIST with extra-adrenal paragangliomas and pulmonary chondromas [10], while Carney-Stratakis syndrome is inherited as an autosomal dominant trait and describes the association of paraganglioma and GIST [11]. The dyad is caused by germline mutations in the *succinate dehydrogenase* (*SDH*) subunits *B*, *C* or *D* (*SDHx*) genes [12]. Absent SDHB expression by immunohistochemistry has been reported in GISTs of Carney triad [13] and indeed WT GIST more broadly [14], however germline mutations of *SDH B, C* or *D* were identified in only 12% of such cases without a family history of paraganglioma [14]. Very recently, it has emerged that mutations of *SDHA* also occur in adult WT GIST and indeed ∼50% of adult WT GISTs contain *SDHx* mutations with ∼70% of these in *SDHA* [15], [16]. By contrast, SDHB is strongly expressed in *KIT*- and *PDGFRA*-mutant GISTs [13], [14].

Given that the oncogenic changes identified at a genomic level in mutant GISTs are not seen in the WT setting, we hypothesized that paediatric and adult WT GISTs are driven in significant part at least by epigenetic and/or post-transcriptional dysregulation. With that in mind, this study was conducted to profile miRNA expression in pediatric and adult WT GIST compared to adult mutant GIST.

## Materials and Methods

### Ethics Statement

Ethical approval was obtained from the Medical Research Ethics Committee, Our Lady’s Children’s Hospital, Crumlin, Dublin 12, Ireland for the use of anonymised, pre-existing (archival) diagnostic material from GIST specimens collected from various European and American sources. The participants all gave written informed consent up-front at diagnosis for inclusion in biological studies of their respective country’s cancer group as a global consent.

### Cases for Study

Samples were collected from European and US collaborators (see acknowledgements). Age categorisation was: <20 years as pediatric and >/ = 20years as adult. Previously genotyped adult mutant GISTs (cases 1–27) were obtained from the archives of MD-R. Additional adult mutant, pediatric and adult WT cases were collected mainly from the NIH pediatric and wild-type GIST clinic, with additional cases accrued from European sources. These cases were mainly gastric, given that pediatric GIST classically arises in the stomach, but also included four small bowel and one retroperitoneal WT case, as this material became available. These cases were all genotyped in the laboratory of MO’S such that the final cohort comprised 30 adult mutant [1.5 male: 1 female; all gastric], 25 adult WT (1 male: 4 female; 20 gastric : 4 small bowel : 1 retroperitoneal) and 18 pediatric WT (1 male: 2 female; all gastric) cases.

### DNA and RNA Extraction

Unstained sections were cut from paraffin blocks at 10 µm (5 per sample) and macro-dissected for tumor tissue only. DNA and RNA extraction was performed using the RecoverAll™ Total Nucleic Acid Isolation Kit for FFPE (Ambion®, Austin, TX, USA), which is known to preserve miRNAs, as per manufacturer’s instructions and quantified using the Nanodrop ND 1000 spectrophotometer (Thermo Scientific, Wilmington, DE, USA).

### Mutational Analysis

Tumor DNA was PCR-amplified for *KIT* exons 9, 11, 13, 17 and *PDGFRA* exons 12, 14, and 18 using previously published primers for *KIT* exons 9, 11 and 13 [17] and newly-designed primers for all other exons tested (Table S1). The PCR products were examined by High Resolution Melt Curve Analysis, with LC Green (Idaho Technology, Salt Lake City UT, USA) and analysed using the Roche LightCycler (Roche, Burgess Hill, West Sussex, UK). Samples with aberrant melt curves were subjected to Sanger sequencing (LGC Genomics GmbH, Berlin, Germany). PCR amplification and sequencing of *BRAF* exon 15 was conducted for adult WT gastric, small bowel and retroperitoneal GISTs using forward primer 5′TGCTTGCTCTGATAGGAAAATG and reverse primer 5′AGCATCTCAGGGCCAAAAAT with an annealing temperature of 59°C.

### MicroRNA Profiling and Data Pre-processing

MiRNA profiling was performed using TaqMan® Low Density Arrays (Applied Biosystems, Foster City, CA, USA). TaqMan® Low Density Arrays allow the profiling and accurate quantitation of 667 miRNAs in a set of two 384-well cards; pool A and pool B, with the cards containing MammU6, RNU24, 43, 44, 48 and 6B as internal controls. Reverse transcription was performed using the TaqMan® MicroRNA Reverse Transcription Kit (Applied Biosystems, Foster City, CA, USA) and MegaPlex™ RT primers (Applied Biosystems, Foster City, CA, USA) pool A and pool B. Products from the reverse transcription were pre-amplified using TaqMan® PreAmp MasterMix and PreAmp primers (pool A and pool B) (Applied Biosystems, Foster City, CA, USA). The array cards were loaded with the preamplified sample and run on the 7900HT Fast Real-Time PCR System (Applied Biosystems, Foster City, CA, USA). Ct values over 35 were considered noise and were disregarded. MiRNAs expressed in less than 20% of samples were excluded from analysis. Mean normalization was carried out by subtracting the mean sample Ct from individual Ct values. Normalized relative expression of miRNAs was calculated with reference to Ct max using: Normalized relative expression = 2^(Ctmax−Ct)^.

### Technical Validation

Three miRNAs, miR-455-5p, miR-488 and miR-124 were used to validate the arrays. Mature miRNA expression levels were evaluated using individual TaqMan® miRNA assay (Applied Biosystems, Foster City, CA, USA) according to the manufacturer’s protocol.

### Data Analysis and Visualization

Cluster Analysis, heatmap generation and boxplots were performed using *hclust*, *heatmap.plus* and *graphics* packages from R statistical programming language v2.8.1. Hierarchical clustering was performed using Spearman’s rank correlation and Ward’s linkage. Heatmap colouring was based on rank of sample value across each miRNA. Statistically significant (p<0.05) changes in miRNA expression levels over various sample classes were calculated using Wilcoxon’s rank-sum test and corrected for multiple comparisons using Bonferroni method. Next, the data were interrogated for miRNAs which showed statistically significant (p<0.05) differential expression between the classes: 1) adult mutant vs. pediatric GIST, 2) adult WT vs. pediatric WT, 3) adult [all] vs. pediatric GIST, 4) adult WT vs. adult mutant GIST, 5) all WT vs. all mutant GIST, 6) cases in cluster B2a vs. B2b (which contains all Carney triad cases, n = 4 known at the time of writing) from the heatmap, and for cases where relevant data were available additional analysis included : 7) SDHB-immunopositive vs. SDHB-immunonegative, 8) 14q loss vs. no loss, and for adult samples alone: 9) high vs. low risk, 10) outcome: died of disease or alive with disease vs. no evidence of disease. As miRNAs function by either degrading target mRNAs or blocking their translation, we were interested in seeking evidence of such interactions. To this end we integrated mRNA expression data and predicted target data (TargetScan [18]–[20]) to establish which sets of miRNA and mRNA were - 1) diametrically expressed across GIST classes and 2) had an over-representation of predicted binding interactions. miRNA : mRNA interactions were examined using our data for differentially expressed miRNAs and pre-existing mRNA expression data [based on data from differentially expressed genes previously published [5], [9], [21]–[28] and indeed full raw, unpublished gene expression data [5]. This analysis involved assessment of the number of predicted interactions (listed in TargetScan [18]–[20]) compared to those expected by chance using *MirMatcher*, a custom-built software application, implemented in Java. The comparisons that were possible included differential mRNA expression between classes: 1) Genes higher in pediatric compared to adult mutant – miRNAs lower in pediatric compared to adult mutant, 2) Genes lower in pediatric compared to adult – miRNAs higher in pediatric compared to adult, 3) Genes higher in mutant compared to WT – miRNAs lower in mutant compared to WT, 4) Genes higher in WT compared to mutant – miRNAs lower in WT compared to mutant, 5) Genes higher in pediatric compared to adult WT – miRNAs lower in pediatric compared to adult WT and 6) Genes higher in pediatric compared to adult mutant – miRNAs lower in pediatric compared to adult mutant and corresponding diametrically expressed miRNAs for the published gene expression data; and classes WT vs. mutant for the unpublished full raw mRNA expression data to which we had access.

### Fluorescence In-Situ Hybridisation [FISH]

FISH analysis for 14q32 loss in adult mutant samples and selected adult WT and pediatric samples, was performed on 4 µm formalin-fixed paraffin-embedded tissue sections as previously described [29]. Ploidy was investigated using double colour locus specific identifier IGH/CCND1 (Applied Biosystems, Foster City, CA, USA), which contains differently labelled probes for IGH/14q32 and CCND1/11q12 genes. Detection was by previously described methods [29].

### Methylation Specific PCR for 14q32

Bisulfite conversion of 1 µg of genomic DNA (where available) was performed with the EpiTect Bisulfite Kit (Qiagen GmbH, Crawley, West Sussex, UK) following the protocol for formalin-fixed paraffin-embedded samples. Methylation specific PCR reactions were performed using ‘maternal’ and ‘paternal’ primers for the differentially methylated 5′ region of the MEG3 promoter as described [30]. Briefly, 50 ng of bisulfite converted DNA was amplified in a 25 µL volume, with 2.5units HotStarTaq DNA Polymerase, 1X Buffer, 200 µM each dNTP (Qiagen GmbH, Crawley, West Sussex, UK), 0.4 µM each methylated primer and 0.8 µM each unmethylated primer. Cycling conditions were 95°C 15 mins, 35 cycles of 94°C 30 s, 58°C 1 min, 72°C 1 min and final extension 72°C 10 mins. PCR products were run on 2.5% agarose gels and post-stained with GelStar Nucleic Acid Stain (Lonza, Muenchensteinerstrasse, Basel, Switzerland). Gels were visualised using a transilluminator.

### Succinate Dehydrogenase B Immunohistochemistry

Where tissue sections from tumors were available, SDHB immunohistochemistry was evaluated using a mouse monoclonal antibody 21A11AE7 (Abcam, Cambridge, MA, USA). The primary antibody dilution was 1∶1000, and immunostaining in a Leica Bond-Max automated immunostainer (Leica Biosystems, Nussloch, Germany) included a step of heat-induced epitope retrieval by a high-pH buffer for 25 min. Primary antibody was incubated for 30 min, and the detection system for 15 min. Diaminobenzidine was used as the chromogen.

### Cell Culture and Transfection

No wild-type GIST cell lines are currently available. The GIST T1 cell line was established by Taguchi [31] from a metastatic GIST of the stomach and harbours a KIT exon 11 mutation. GIST T1 cells were a generous gift from Dr. Jonathan Fletcher (Dana-Farber Cancer Institute, Boston, MA, USA) and were cultured in Iscove’s Modified Dulbecco’s Medium (IMDM) supplemented with 1% penicillin/streptomycin and 10% fetal bovine serum (FBS). Cells were maintained at 37°C in a humidified incubator with 5% CO_2_.

miRNA precursors and a scrambled oligonucleotide control (Ambion®, Austin, TX, USA) were transiently transfected into cells using siPORT NeoFX (Ambion®, Austin, TX, USA) as per manufacturer’s instructions.

### Cell Proliferation Assay

Cell proliferation was assessed using the CellTitre 96 AQ_ueous_ Non-Radioactive Proliferation Assay (MTS) (Promega Corporation, Madison, WI, USA). Cells were transfected with miRNA precursors (miR-34c-5p, miR-185 and miR-190), scrambled oligonucleotide control (SCR) or siKinesin (KIN), a positive control for decreased cell proliferation, and seeded into 96 well plates at a density of 2×10^3^ cells per well. Proliferation was measured every 24 hours over a 96 hour period by reading the plates at 495 nm, using the Synergy Mx Monochromator-Based Multi-Mode Microplate Reader (BioTek, VT, USA).

### Scratch Assay

Cells were transfected as described with miRNAs miR-34c-5p, miR-185 and miR-190, and plated at a density of 2.4×10^5^ to allow for confluency at 48 hours. Forty-eight hours after transfection, confluent cells were scratched with a P1000 sterile tip, held perpendicular to the plate. Media was removed and cells washed twice with pre-warmed PBS and once with pre-warmed media. Fresh media was then added to the cells and they were photographed (0 hours) using the Olympus UC30 camera attached to an Olympus CKX41SF inverted microscope (Olympus, Tokyo, Japan). Cells were again washed at 24 and 48 hours post-scratch and photographed at these time-points also. All images were compared to the SCR control.

### Quantitative Real-Time PCR

miRNAs were extracted from the cells using the miRNeasy miRNA mini Kit (Qiagen GmbH, Crawley, West Sussex, UK) and quantitative Real-Time PCR was carried out as described above.

## Results

### Mutational Analysis

Mutational analysis showed mutually exclusive *KIT* mutations in 18/73, *PDGFRA* in 11/73 cases and a *BRAF* mutation in a single case. The remaining 43 cases were WT for the exons tested in these genes. All clinical and genomic data are provided in Tables 1, 2, 3.

**Table 1:** Adult Mutant GIST case demographics.

Case	Sex	Age	Location	Genotype	Mutation	14q32 Status	SDHB IHC	Follow-up	Risk	Histology
**1**	F	58	Stomach	KIT ex 11	Y553_W557del	Loss	NA	DOD	H	S
**2**	F	37	Stomach	PDGFRA ex 18	D842V	Loss	NA	WD	L	S
**3**	M	55	Stomach	PDGFRA ex 14	N659K	Loss	NA	WD	L	E
**4**	F	73	Stomach	PDGFRA ex 18	D842V	Loss	NA	WD	L	E
**5**	F	54	Stomach	KIT ex 11	M552_W557del	Loss	NA	WD	I	S&E
**6**	M	44	Stomach	KIT ex 11	W557R	Loss	NA	WD	I	S&E
**7**	F	78	Stomach	KIT ex 11	P551_E561delinsLQ	Diploid	NA	AWD	H	S
**8**	F	65	Stomach	KIT ex 11	W557_V560delinsF	Loss	NA	AWD	H	E
**9**	M	85	Stomach	KIT ex 11	V559A	Loss	NA	WD	L	S&E
**10**	M	62	Stomach	PDGFRA ex 18	D842V	Loss	NA	WD	H	E
**11**	M	44	Stomach	PDGFRA ex 18	D842V	Loss	NA	WD	I	S
**12**	M	70	Stomach	KIT ex 11	W557_K558del	Loss	NA	AWD	H	E
**13**	M	77	Stomach	KIT ex 11	V559D	Loss	NA	WD	L	E&S
**14**	F	27	Stomach	KIT ex 11	556_573del	Loss	NA	AWD	I	S
**15**	M	71	Stomach	KIT ex 11	V560_G565del	Loss	NA	AWD	H	S
**16**	M	72	Stomach	PDGFRA ex 18	D842_M844del	Loss	NA	WD	I	E
**17**	M	72	Stomach	KIT ex 11	K558_V559delinsN	Loss	NA	AWD	H	S
**18**	M	62	Stomach	KIT ex 11	V554D	Loss	NA	WD	I	S
**19**	M	67	Stomach	KIT ex 11	W557_K558del	Diploid	NA	DOD	H	S
**20**	M	69	Stomach	PDGFRA ex 18	D842_I843del	Loss	NA	WD	I	S
**21**	M	74	Stomach	KIT ex 11	V560D	Loss	NA	DOD	H	E
**22**	F	48	Stomach	KIT ex 11	K550_V555del	Loss	NA	WD	H	S
**23**	M	49	Stomach	PDGFRA ex 18	D842V	Trisomy	NA	WD	H	E
**24**	M	68	Stomach	PDGFRA ex 12	V561D	Loss	NA	WD	I	E&S
**25**	M	50	Stomach	PDGFRA ex 18	D842V	Loss	NA	WD	L	E
**26**	F	71	Stomach	KIT ex 11	W557_K558del	Loss	NA	AWD	H	E
**27**	F	46	Stomach	PDGFRA ex 18	D842V	Diploid	NA	WD	I	E
**50**	F	58	Stomach	KIT ex 9	S476I	NA	positive	NA	NA	E&S
**51**	F	22	Stomach	KIT ex 11	Y553_W557del	NA	positive	NA	NA	E
**52**	M	58	Stomach	BRAF ex 15	V600E	Diploid	positive	NA	NA	E&S

NA – Not Available, DOD – Died of Disease, WD – Without Disease, AWD - Alive with Disease, WT – Wild-Type, S – Spindle, E - Epithelioid.

Patient demographics including age, sex, syndromic association and outcome, as well as data pertaining to tumors including anatomic location, histological type, KIT/PDGFRA/BRAF mutational status, 14q status, risk and SDH immunoreactivity where available.

**Table 2:** Adult WT case demographics.

Case	Sex	Age	Location	Genotype	Mutation	14q32 Status	SDHB IHC	Follow-up	Risk	Histology
**28**	F	24	Stomach	WT	WT	NA	NA	NA	NA	E
**29**	F	22	Stomach	WT	WT	NA	NA	NA	NA	E
**30**	F	20	Stomach	WT	WT	NA	NA	NA	NA	E
**32**	F	22	Stomach	WT	WT	NA	NA	NA	NA	E
**35**	F	23	Stomach	WT	WT	NA	negative	NA	NA	E
**36**	F	21	Stomach	WT	WT	Diploid	negative	NA	NA	E
**38**	F	25	Stomach	WT	WT	Diploid	negative	NA	NA	E
**46**	M	22	Stomach	WT	WT	NA	negative	NA	NA	E
**48**	M	20	Stomach	WT	WT	NA	negative	NA	NA	E
**49**	F	46 [26]	Stomach	WT	WT	NA	negative	NA	NA	NA
**53**	F	20	Jejunum	WT	WT	Diploid	positive	NA	NA	E&S
**54**	F	78	Stomach	WT	WT	NA	NA	NA	NA	E
**55**	F	50	Stomach	WT	WT	NA	NA	NA	NA	E
**56**	M	42	Stomach	WT	WT	NA	NA	NA	NA	S
**60**	F	33	Stomach	WT	WT	NA	positive	NA	NA	S
**63**	F	30	Jejunum	WT	WT	Diploid	positive	NA	NA	S
**64**	F	40	Stomach	WT	WT	NA	negative	NA	NA	E
**65**	F*	28	Stomach	WT	WT	NA	negative	NA	NA	E
**66**	F	54	Ileum	WT	WT	Diploid	positive	NA	NA	S
**68**	F	55	Stomach	WT	WT	NA	negative	NA	NA	E
**69**	F	49	Stomach	WT	WT	NA	negative	NA	NA	E
**70**	F	31	Stomach	WT	WT	NA	negative	NA	NA	E
**71**	M	45	Ileum	WT	WT	Trisomy	positive	NA	NA	E
**72**	F	46	Stomach	WT	WT	NA	positive	NA	NA	S
**73**	M	37	Retroperitoneum	WT	WT	Diploid	positive	NA	NA	S

Patient demographics including age, sex, syndromic association and outcome, as well as data pertaining to tumors including anatomic location, histological type, KIT/PDGFRA/BRAF mutational status, 14q status, risk and SDH immunoreactivity where available.

**Table 3:** Pediatric GIST case demographics.

Case	Sex	Age	Location	Genotype	Mutation	14q32 Status	SDHB IHC	Follow-up	Risk	Histology
**31**	F	15	Stomach	WT	WT	NA	NA	NA	NA	E
**33**	M	13	Stomach	WT	WT	NA	NA	NA	NA	E
**34**	F	14	Stomach	WT	WT	NA	NA	NA	NA	E
**37**	M	14	Stomach	WT	WT	Diploid	negative	NA	NA	S&E
**39**	F	10	Stomach	WT	WT	Diploid	negative	NA	NA	S&E
**40**	F	17 [10]	Stomach	WT	WT	Diploid	negative	NA	NA	E
**41**	M*	14	Stomach	WT	WT	Diploid	negative	NA	NA	E
**42**	F*	17 [12]	Stomach	WT	WT	Diploid	negative	NA	NA	E
**43**	F	10	Stomach	WT	WT	NA	NA	NA	NA	E
**44**	F*	16	Stomach	WT	WT	NA	NA	NA	NA	E
**45**	F	8	Stomach	WT	WT	NA	negative	NA	NA	E
**47**	M	18	Stomach	WT	WT	NA	negative	NA	NA	E
**57**	M	10	Stomach	WT	WT	NA	negative	NA	NA	E
**58**	F	12	Stomach	WT	WT	NA	NA	NA	NA	NA
**59**	F	11	Stomach	WT	WT	NA	negative	NA	NA	E
**61**	M	18	Stomach	WT	WT	NA	NA	NA	NA	E
**62**	F	19	Stomach	WT	WT	NA	negative	NA	NA	E
**67**	F	18	Stomach	WT	WT	NA	negative	NA	NA	E

NA – Not Available, DOD – Died of Disease, WD – Without Disease, AWD - Alive with Disease, WT – Wild-Type, S – Spindle, E – Epithelioid, * Carney triad.

Patient demographics including age, sex, syndromic association and outcome, as well as data pertaining to tumors including anatomic location, histological type, KIT/PDGFRA/BRAF mutational status, 14q status, risk and SDH immunoreactivity where available.

### MicroRNA Profiling

**Figure 1:**
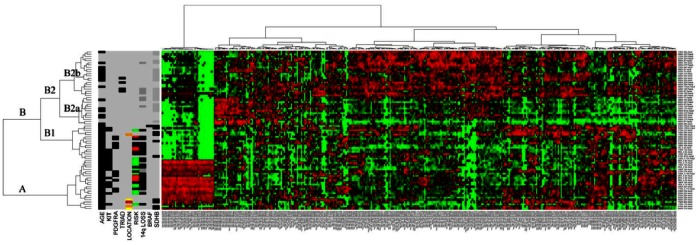
Full heatmap generated from complete miRNA expression data for all 73 cases. Included at the left of heatmap are case data to include adult/pediatric categorisation, anatomic location of tumor, *KIT*, *PDGFRA* and *BRAF* mutational status, risk stratification for adult mutant cases, and, where available: Carney triad status, SDHB-immunoreactivity and 14q genomic status of tumor. Black = positive for feature listed. For age, black = adult, grey = pediatric; for *KIT/PDGFRA/BRAF* black = mutation positive while grey = WT; Black = Carney triad diagnosed; for anatomic location of tumor, grey = stomach, red = retroperitoneum, yellow = jejunum; orange = ileum; for risk, red = low, black = intermediate, green = high, grey = unavailable. For 14q loss and SDHB status, black = positive; dark grey = tested and negative, grey = not tested. The clusters are designated A, B, with sub-clusters B1 and B2, B2a and B2b to facilitate discussion of findings.

**Figure 2:**
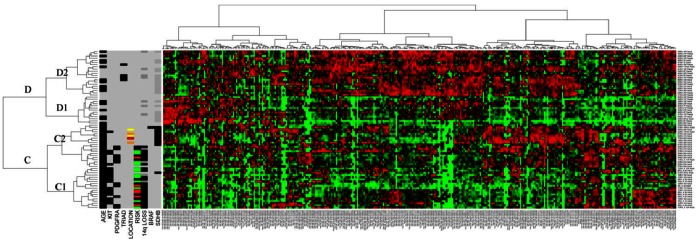
Heatmap minus dominant 14q miRNA expression. The clusters are designated C and D with sub-clusters C1, C2, D1 and D2 to facilitate discussion of findings. Case data as delineated for figure 1. Black = positive for feature listed. For age, black = adult, grey = pediatric; for *KIT/PDGFRA/BRAF* black = mutation positive while grey = WT; Black = Carney triad diagnosed; for anatomic location of tumor, grey = stomach, red = retroperitoneum, yellow = jejunum; orange = ileum; for risk, red = low, black = intermediate, green = high, grey = unavailable. For 14q loss and SDHB-immunoreactivity status, black = positive; dark grey = tested and negative, grey = not tested. The clusters are designated A, B, with sub-clusters B1 and B2, B2a and B2b to facilitate discussion of findings.

Unsupervised hierarchical clustering based on all miRNAs showed a separation into clusters A and B (Figure 1), with cluster B subdivided into B1 and B2. Adult mutant cases are located in Clusters A and B1 and pediatric GISTs in Cluster B2, with adult WT GISTs dispersed amongst both adult mutant and pediatric WT cases (Figure 1). The clear split within the adult mutant cohort into clusters A and B1 is due to differential expression of forty-seven miRNAs located on chromosome 14q32.2 and 14q32.31. Following removal of the dominant 14q miRNA cluster from the heatmap, the split between adult mutant and pediatric WT GISTs is accentuated with the samples split into clusters C and D, such that adult mutant cases are in cluster C and pediatric WT cases in cluster D (Figure 2). Both these clusters can be further subdivided into C1, C2, D1 and D2 (Figure 2). The adult WT cases remain dispersed amongst both adult mutant and pediatric WT cases on this modified heatmap and the WT small bowel and retroperitoneal GISTs cluster tightly together in Cluster C2 with adult mutant cases (Figure 2). In both these situations, the SDHB status underpins the clustering, such that the SDHB-immunopositive WT gastric, small bowel and retroperitoneal cases cluster with the adult mutant cases, while SDHB-immunonegative adult WT cases cluster with the pediatric WT cases. Sixteen miRNAs were found to be significantly differentially expressed between SDHB positive and SDHB negative cases. Some of the miRNAs with recently identified targets *in vitro* in various tumor settings other than GIST include: miR-132 targeting *Rb1* [32], miR-193b targeting *CCND1* and *Mcl-1* [33], miR-455-3p targeting *Smad2* [34], miR-125b targeting *Mcl-1* and *Bcl-2* [35] and miR-542-5p targeting *survivin* [36]. However to-date, none of these miRNAs has been found to target any of the subunits of SDH.

### Technical Validation of miRNA Array

The miRNAs selected for validation of the TaqMan® low density array cards (Applied Biosystems, Foster City, CA, USA) were miR-455-5p which was significantly upregulated in mutant GIST (p<0.00003) and miRNAs miR-488 and miR-124, which were significantly upregulated in WT GISTs (p<0.05), showing good concordance with the array results.

### Pair-wise Comparisons of miRNA Expression between Classes of GISTs

Apart from the comparison of adult WT vs. pediatric WT, all other paired comparisons highlighted significant differences in miRNA expression (Table 4). Review of the putative targets of the miRNAs, using TargetScan [18]–[20], showed that many are predicted to target genes of known biological importance in GIST, including *KIT*, *PDGFRA*, *IGF1R* and *MAPK1*.

**Table 4:** Significantly differentially expressed miRNAs between the various classes of GIST.

Class 1	Class 3	Class 4	Class 5	Class 6	Class 7
**Adult Mutant vs.** **Pediatric**	**Adult vs.** **Pediatric**	**Adult WT vs.** **Adult Mutant**	**ALL WT vs.** **ALL Mutant**	**B2a vs. B2b**	**SDHB+ vs. SDHB−**
**miRs higher in** **adult mutant**	**miRs higher** **in adult**	**miRs higher** **in adult WT**	**miRs higher** **in ALL WT**	**miRs higher** **in B2b**	**miRs higher** **in SDHB+**
let-7f-2*	miR-193b	miR-625	miR-422a	let-7d	miR-132
**miR-139-5p**	miR-455-3p	miR-638	miR-423-5p	let-7g	miR-146a
**miR-152**	miR-455-5p	miR-744	miR-450a	let-7e*	miR-193b
miR-181a-2*		miR-923	miR-450b-5p	miR-7-1*	miR-193b*
miR-193b*	**miRs higher** **in pediatric**		miR-452	miR-17	miR-455-3p
miR-193b*	miR-125b-1*	**miRs higher** **in adult mutant**	miR-488	miR-20b	miR-455-5p
**miR-340**	miR-186	let-7f-2*	miR-488*	miR-26b*	miR-484
**miR-365**	miR-488	miR-365	miR-491-5p	miR-26-b-2*	miR-886-5p
**miR-455-3p**	miR-551b*	miR-455-3p	miR-523	miR-30b	
**miR-455-5p**	miR-576-3p		miR-542-5p	miR-30c	**miRs higher in SDHB-**
**miR-886-3p**		**Class 5**	miR-548b-5p	miR-93*	miR-125b
**miR-886-5p**	**Class 4**	**ALL WT vs.** **ALL Mutant**	miR-551b*	miR-106a	miR-450b
	**Adult WT vs.** **Adult Mutant**	**miRs higher** **in ALL WT**	miR-576-3p	miR-125a-5p	miR-488
**miRs higher in** **pediatric GIST**	**miRs higher** **in adult WT**	let-7d	miR-590-5p	miR-126	miR-488*
miR-7-2*	miR-16	miR-16	miR-625	miR-132	miR-542-3p
**miR-15a**	miR-20b	miR-20b	miR-638	miR-191	miR-551b
**miR-16**	miR-28-3p	miR-28-3p	miR-873	miR-212	miR-576-3p
**miR-34c-5p**	miR-34c-5p	miR-34c-5p	miR-923	miR-331-3p	miR-769-5p
**miR-125a-3p**	miR-124	miR-124		miR-339-3p	
miR-125b-1*	miR-125a-3p	miR-125a-3p		miR-340*	
**miR-126**	miR-126*	miR-125b-1*	**Class 5**	miR-342-3p	**Class 8**
**miR-129-3p**	miR-126	miR-126	**miRs higher** **in ALL Mutant**	miR-361-5p	**14q Loss vs.** **No Loss**
**miR-186**	miR-129.3p	miR-126*	miR-let-7f-2*	miR-362-3p	**miRs higher with no 14q Loss**
**miR-190**	miR-129.5p	miR-129-3p	miR-152	miR-363	miR-20b
**miR-192**	miR-155	miR-129-5p	miR-154	miR-374a	
**miR-210**	miR-181a-2*	miR-155	miR-193b	miR-374b	
**miR-214**	miR-185	miR-181a-2*	miR-193b*	miR-484	
**miR-345**	miR-186	miR-184	miR-302b	miR-532-3p	
**miR-361-5p**	miR-197	miR-185	miR-324-5p	miR-598	
**miR-383**	miR-202	miR-186	miR-365	miR-671-3p	**Class 9**
**miR-422a**	miR-210	miR-192	miR-377*	miR-708	**High vs. Low Risk**
**miR-423-5p**	miR-214	miR-197	miR-410	miR-744*	**miRs higher in low risk**
**miR-450a**	miR-331-5p	miR-202	miR-455-3p		miR-150
**miR-450b-5p**	miR-339-3p	miR-210	miR-455-5p	**miRs higher in B2a**	
miR-488*	miR-345	miR-214	miR-668	miR-572	
**miR-488**	miR-361-5p	miR-331-5p	miR-744		**Class 10**
**miR-491-5p**	miR-422a	miR-338-3p			**DOD/AWD vs. NED**
**miR-523**	miR-450a	miR-339-3p			**miRs higher in DOD/AWD**
**miR-542-5p**	miR-452	miR-340			miR-19b
miR-551b*	miR-523	miR-342-3p			
**miR-576-3p**	miR-542-5p	miR-345			
**miR-590-5p**	miR-548b-5p	miR-361-5p			
**miR-744**	miR-548c-3p	miR-363			
**miR-873**	miR-548d-5p	miR-383			

The various pair-wise comparisons between classes of GIST are listed with miRNAs significantly differentially expressed between these.

### Bioinformatic Evaluation of miRNA: mRNA Interactions

The data were interrogated for likely significant biological interactions between diametrically expressed miRNA and mRNA as described above. To determine if any miRNAs were predicted to target these inversely expressed mRNAs we used the TargetScan database of predicted interactions [18]–[20]. In the comparison of significantly up-regulated mRNAs: down-regulated miRNAs in the pediatric *versus* adult mutant classes, we observed a significantly (p<0.006) higher degree of predicted interactions than expected. These bioinformatic data suggest that the differential expression of these mRNAs may be due in some part to post-transcriptional regulation by these differentially expressed miRNAs. These interactions included *ANK3*, *IGF1R, NLGN4X, FZD2* and *PHKA1* with miR-139-5p, miR-152, miR-193b, miR-340 miR-365, 455-5p, 455-3p, 886-3p and 886-5p. From the full raw gene expression dataset [5], only for the comparison where gene expression was higher and miRNAs lower in mutant *versus* WT GIST, were the predicted mRNA: miRNA interactions significantly (p<0.0121) over-represented, suggesting that these interactions might be functionally relevant. These included interactions of miR-509-5p, 330-3p, 455-5p, 152, 193b, 302b and 365 with *IGF1R, PPARGC1A* and *PRDM16*.

### Fluorescence In-Situ Hybridisation (FISH)

FISH analysis for 14q32 was performed on 28 adult mutant samples, with 82% of cases showing 14q32 loss. FISH analysis for 14q32 in 2 adult WT samples and 5 pediatric samples all showed a diploid phenotype at 14q32 (Tables 1, 2, 3).

### Methylation Analysis of 14q32 Region

**Figure 3:**
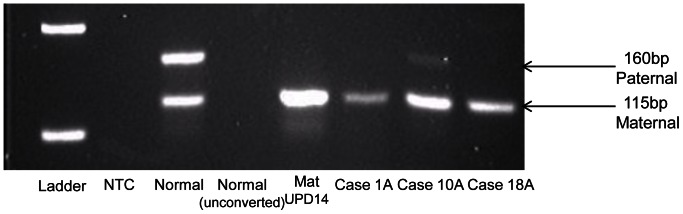
Loss of Paternal 14q32 Allele in Cluster A. Agarose gel for cases in Cluster A showing loss of the paternal allele. Lane 1: Molecular marker, 2: NTC-no template control, 3: Normal sample, 4: Normal sample unconverted, 5: positive maternal UPD14, Lanes 6–8: adult mutant cases.

**Figure 4:**
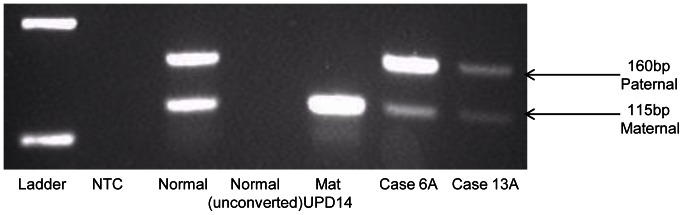
Loss of Maternal 14q32 Allele in Cluster B1. Agarose gel for cases in Cluster B1 showing loss of the maternal allele. Lane 1: Molecular marker, 2: NTC-no template control, 3: Normal sample, 4: Normal unconverted, 5: positive maternal UPD14, 6–7: adult mutant cases.

While 14q loss is a common genomic observation with disease progression in adult mutant GIST, it is immediately obvious from the heatmap annotations, that there is no direct correlation between 14q miRNA expression and 14q genomic status (in cases where it was possible to investigate 14q loss by FISH). Eighty-two percent of adult mutant cases tested show 14q32 loss (Table 1). This is a known imprinted region on 14q32 and all miRNAs in this region are derived from one transcript which is maternally expressed [37], [38]. We hypothesised that differential allelic losses in this imprinted region might explain the observed miRNA expression patterns. In other words, loss of 14q32 involving the paternal allele would effectively be silent and so, despite evidence of genomic loss by FISH, the maternal expressed allele would be retained, explaining expression of the miRNAs on the heatmap for such cases. We tested this by examining the methylation status of the 14q region. Preferential loss of the paternal [silent] allele was observed in 75% [9/12, of which 2 = adult WT] of cases tested from Cluster A, where there is relative preservation of 14q miRNA expression (Figure 3), while the expressing maternal allele is preferentially lost in 83% [5/6, of which 2 = adult WT] of cases tested from Clusters B1 (Figure 4). Importantly, the remaining cases tested from clusters A and B1 all showed normal methylation patterns. The pediatric cases tested show normal methylation patterns in conjunction with an absence of genomic losses of the 14q region, such that their relatively lower expression of 14q miRNAs in this cohort must be accounted for by some other mechanism.

From the full heatmap (Figure 1) Cluster B2 can be further subdivided into Cluster B2a and B2b. Note that all known Carney triad patients (n = 4) fall into Cluster B2b.

### SDHB Immunohistochemistry

SDHB immunohistochemistry was performed on 32/73 samples for which slides were available. Eleven adult WT and 11 pediatric WT cases were negative for SDHB and 7 adult WT and 3 adult mutant cases were positive for SDHB (Tables 1, 2, 3).

### Cell Proliferation and Scratch Assay

**Figure 5:**
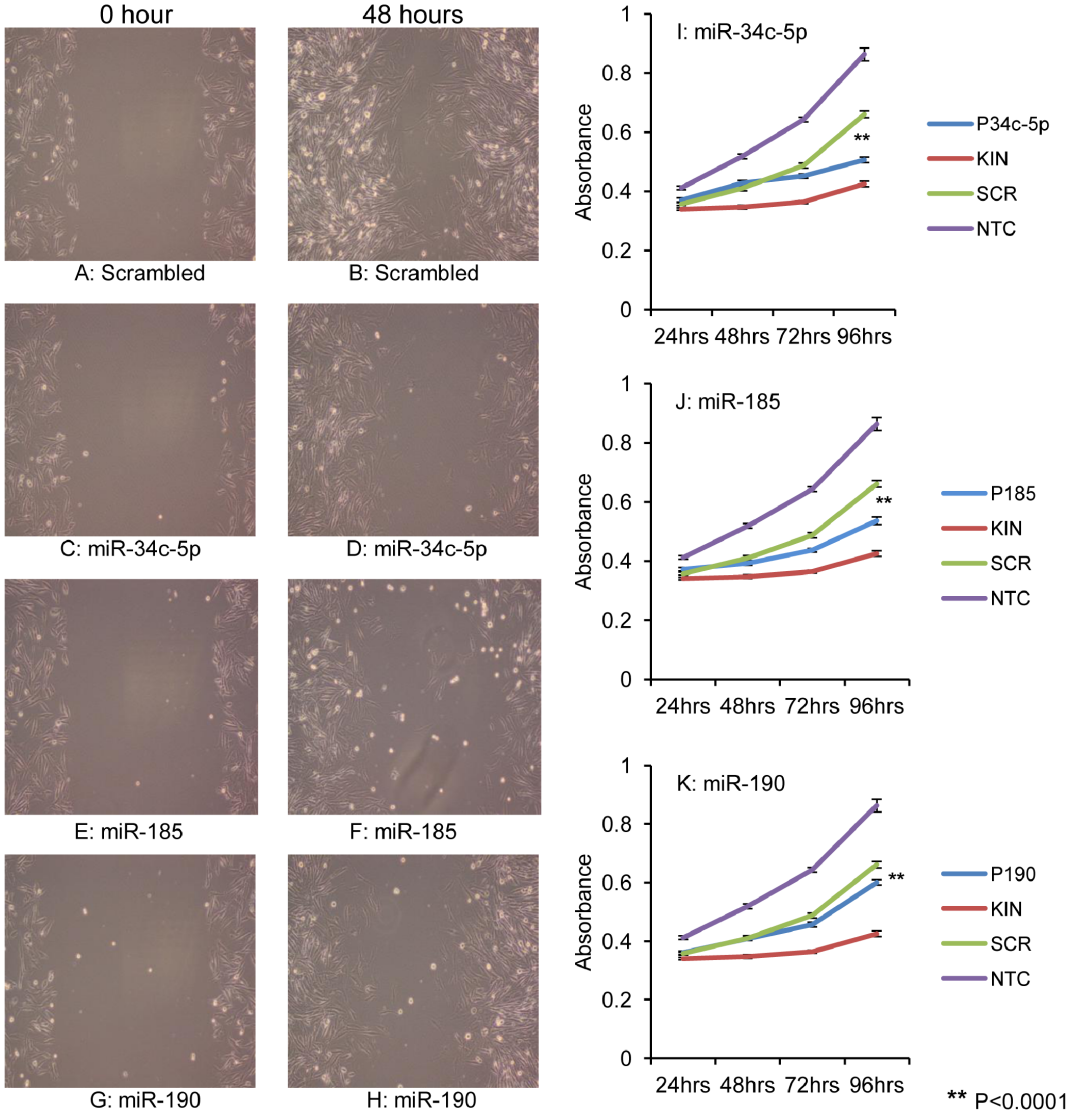
Functional studies of selected differentially expressed miRNAs. Transfection with miR-34c-5p, miR-190 and miR-185, (all of which are expressed at higher levels in pediatric compared with adult mutant cases), show *decreased* wound healing compared to the SCR control 48 hours after scratch assay (Figures A–H). Ectopic expression of miR-34c-5p, miR-190 and miR-185 showed a significant *decrease* in cell proliferation at 96 hours, as measured using an MTS assay, compared to the SCR control (Figures I–K). KIN = siKinesin (positive control for reduced cell proliferation), SCR = scrambled oligonucleotide control, NTC = non-transfected control.

Transfection of GIST T1 cells with mature miRNA mimics miR-34c-5p, miR-190 and miR-185, all of which are expressed at higher levels in pediatric (34c-5p and 185 also in adult WT) compared with adult mutant cases, showed a decrease in wound healing compared to the scrambled control (Figure 5A–H). While miR-34c-5p, miR-185 and miR-190 furthermore demonstrated significantly (P<0.001) decreased cell proliferation compared the scrambled control (Figure 5I–K).

## Discussion

Prompted by the lack of data from genomic studies to explain pediatric/WT GIST oncogenesis, we undertook this study of miRNA profiling to investigate a role for post-transcriptional dysregulation by these non-coding RNAs in pediatric/WT GIST development. Unsupervised hierarchical clustering split the cases into two large groups, Cluster A and Cluster B, resulting from differential expression of forty-seven miRNAs located on 14q32.2 and 32.31. Two prior studies [39], [40] showed similar differential miRNA expression patterns in adult mutant GIST based on 14q status, as well as other clinico-pathological variables. However neither of those studies addressed the methylation status of the retained 14q allele in their cases showing 14q loss. We found no direct relationship between 14q genomic status and 14q32 miRNA expression in this cohort. Eighty-two percent of adult mutant cases tested showed 14q loss, yet many of these in fact show relatively higher 14q miRNA expression than cases with the normal (diploid) FISH result as seen in all pediatric cases. The 14q32 region is a known imprinted region in both mice (where the corresponding region is located on distal chromosome 12) and humans [37], [38], [41], containing maternally- and paternally-expressed genes. The miRNAs located within this cluster all map within a 40 kb interval and are controlled by a differentially methylated region (IG-DMR) 200 kb away [37], [38], [42]. miRNAs in this region are only expressed from the maternal allele [37], [38], as the paternal allele is silenced by methylation, and these miRNAs are thought to be transcribed as a large single poly-cistronic cluster (precursor transcript) rather than as individual primary transcripts [37]. Therefore, deletion of the active maternal allele is required for complete loss of expression of these miRNAs. We hypothesised that the adult mutant cases showing 14q loss with relatively higher 14q miRNA expression (Cluster A) must retain the active maternal allele, while the cases with lower 14q miRNA expression (Cluster B) retain the silent paternal allele, resulting in down-regulation of these miRNAs. To investigate this, we applied the diagnostic assay used for the detection of uniparental disomy (UPD) for chromosome 14q [30]. UPD is the inheritance of both homologues of a chromosome from one parent [43]. 14q32 contains the respectively maternally- and paternally- expressed *MEG3* and *DLK1* genes, which contribute to different phenotypes in maternal and paternal UPD14 [30], [41], [43] and are regulated by a differentially methylated region (DMR) that extends over the *MEG3* promoter. This is the IG-DMR referred to above which controls the miRNA cluster. The assessment for UPD14 relies on a methylation-specific multiplex PCR to amplify methylated and unmethylated elements of the DMR and identify normal pattern methylation, maternal or paternal UPD14 [30]. Our results suggest that the adult cases do fit our hypothesis, with 75% (9/12) of cases that were tested from Cluster A showing convincing loss of the paternal allele (Figure 3), while the remaining 3 cases showed a normal pattern; and 83% (5/6) of cases tested from Cluster B1 showing loss of the maternal allele (Figure 4) with the last case showing a normal pattern of methylation. We explain the lack of a perfect correlation between the methylation status of the 14q region and miRNA expression by likely admixture of DNA from non-tumor tissue elements retaining the normally imprinted arrangement. Our hypothesis does not hold true for the pediatric WT cases, with the majority of cases tested showing normal methylation patterns, suggesting that some other mechanism must underlie the down-regulation of the miRNAs in this region in this setting. Further investigation is required to elucidate this.

Many miRNAs are categorised into clusters, often located at fragile sites [44]. The forty-seven miRNAs located on 14q32 represent one of the largest clusters identified to-date [45] and have been found down-regulated or silenced in many cancers including gliomas [45] and epithelial ovarian cancers [46], suggesting a possible tumor suppressor role for the miRNAs located in this cluster. Few of the 14q32 miRNAs have been studied *in vitro* with target validation. Of those differentially expressed in our study, 14q32 miRNAs having confirmed targets include miR-127 (located within a CpG island and silenced in many cancers) targeting *BCL-6* [47], miR-154 targeting *CCND2* [48], miR-433 targeting *HDAC6* [49], miR-485-3p targeting *NF-YB* [50] and miR-539 targeting *HLCS* [51]. Using target prediction algorithms, including TargetScan, [18]–[20], it emerges that these miRNAs may target genes of known importance in GIST cell biology including *KIT*, *PDGFRA IGF1R*, *NF1*, *MAPK1*, *SDH* and *KRAS* [9], [14], [28], however further investigation is required to confirm this as well as other potentially important gene targets. Recently miR-494 was shown to target *KIT* in the GIST882 cell line [52]. Up-regulation of miR-494 in GIST882 cells reduced the expression of *KIT* and its downstream signalling, increased apoptosis and inhibited cell growth, suggesting the possible importance of loss of miR-494 and perhaps indeed all 47 miRNAs on 14q32 in GIST progression [52].

On the heatmaps (Figures 1; 2), the pediatric cases consistently cluster separately from the adult mutant cases. As described, our functional assays suggest that the differentially expressed miRNAs producing this clustering indeed have biological effects on GIST cell lines. The miRNAs chosen here have proven targets in other neoplasms. miR-34c-5p targets *E2F3, MYCN, Bcl-2* and *c-Met* [53], [54], while miR-185 targets RhoA and Cdc42 [55]. Ectopic expression of miR-34c-5p in cervical cancer cells inhibited proliferation and anchorage independent growth, while ectopic expression of miR-185 in colorectal cancer cells also inhibited proliferation, induced G1 cell cycle arrest and apoptosis, and blocked invasion, suggesting tumor suppressive roles for both these miRNAs [53]–[55]. Their lower expression in adult mutant GISTs might contribute to the more aggressive course of these tumors compared with their WT counterparts where expression is higher.

Cluster B2 consists of all pediatric and sixteen adult WT gastric cases which can be further split into Clusters B2a and B2b. All known Carney triad cases cluster together in B2b, which could also contain cases not yet definitively diagnosed as Carney triad at the time of writing (n = 4). In the dyad, paraganglioma is typically the first tumor manifestation, with GIST following, however in Carney triad, generally GIST is the first tumor diagnosed and it can take many years for other tumors [pulmonary chondroma and paraganglioma] to develop, so that the diagnosis of Carney triad may be delayed by years or even decades. The miRNAs differentially expressed between Clusters B2a and B2b therefore may represent a signature that could prompt closer patient monitoring for development of additional tumor manifestations of Carney triad compared to those patients in Cluster B2a. A larger cohort with retrospective clinical data is required for confirmation of such a theory. Thirty-four miRNAs are differentially expressed between the Clusters B2a and B2b. Some of these, including let-7g, miR-212 and miR-132 are known to target *c-MYC* [56], *Rb1* [32] and *RASA1* [57] respectively, in various other tumors.

The heatmap following removal of the dominant 14q cluster (Figure 2) still clearly shows that the adult WT cases are dispersed between adult mutant and pediatric cases and this is apparently on the basis of SDHB reactivity. It has emerged recently that SDHB immunoreactivity is tightly correlated with mutational status in GIST such that mutant cases are consistently SDHB-immunopositive while WT cases are frequently SDHB-immunonegative, notably in the pediatric setting [13], [14]. A result which parallels ours, has recently been identified by Killian *et al* [58] showing that WT GISTs demonstrating a “methyl centrist” profile (a methylation pattern which resembles that of normal tissues) were all positive for SDHB immunohistochemically and had clinical characteristics similar to mutant GISTs with greater tyrosine kinase inhibitor response, while WT GISTs within the “methyl divergent” group (cases showing an outlier methylation pattern relative to normal tissues and mutant GIST samples) were all negative for SDHB by immunohistochemistry and behaved clinically similarly to pediatric GISTs. Both of these epigenetic studies –the Killian *et al* methylation data and our miRNA profiling -have therefore separated adult WT GISTs based on their SDHB immunohistochemistry status, placing SDHB-immunopositive WT GISTs with adult mutant GISTs, a finding which raises the tantalising possibility that these tumors bear yet-to-be identified oncogenic kinase mutations. Such a pattern is observed not only for *gastric* adult WT GISTs, but particularly also for those cases arising in small bowel and retroperitoneum in our cohort.

The more meaningful categorisation of GIST emerging on multiple levels at this stage then, is one based on SDHB immunoreactivity, with the implication that oncogenesis is driven by impaired cellular respiration in the SDHB-immunonegative cases [13], [14], [59].

In summary then, this study has shown very discrete clustering of subsets of GIST based on miRNA profiles with evidence of some functional effects of these differentially expressed miRNAs, excellent clinico-pathological correlations and implications for post-transcriptional dysregulation in pediatric/WT GIST oncogenesis.

## Supporting Information

Table S1 **Unpublished primers used in this study.** Sequences of primers used for mutational analysis of *KIT* exon 17 and *PDGFRA* exons 12, 14, 18 indicating outer and semi-nested primers.
(DOC)
