# Supplementary material for: Post-Transcriptional Dysregulation by miRNAs Is Implicated in the Pathogenesis of Gastrointestinal Stromal Tumor [GIST]
Source: PLoS One. 2013 May 24;8(5):e64102. doi: 10.1371/journal.pone.0064102 (PMC3663836; doi:10.1371/journal.pone.0064102)
Supplement: Table S1 — Unpublished primers used in this study. Sequences of primers used for mutational analysis of KIT exon 17 and PDGFRA exons 12, 14, 18 indicating outer and semi-nested primers. (DOC) [file pone.0064102.s001.doc]

**Table S1**

***KIT* exon 17**

F 5’TTCACTCTTTACAAGTTAAAATG

R1 5’TCACAGGAAACAATTTTTATCGAA (outer)

R2 5’TGCAGGACTGTCAAGCAGAG (semi-nested)

***PDGFRα* exon 12**

F1 5’TCCAGTCAGTGTGCTGCTTC (outer)

R 5’GGAGGTTACCCCATGGAACT

F2 5’CTCTGGTGCACTGGGACTTT (semi-nested)

***PDGFRα* exon 14**

F 5’ TGGTAGCTCAGCTGGACTGAT

R1 5’TTAGGAGGTGAGAGGTAGGG (outer)

R2 5’GGGATGGAGAGTGGAGGATT (semi-nested)

***PDGFRα* exon 18**

F1 5’ACCATGGATCAGCCAGTCTT (outer)

R 5’CTGACCAGTGAGGGAAGTGAG

F2 5’ CAGCTACAGATGGCTTGATCC (semi-nested)
